# Non-prescription dispensing of antibiotic agents among community drug retail outlets in Sub-Saharan African countries: a systematic review and meta-analysis

**DOI:** 10.1186/s13756-020-00880-w

**Published:** 2021-01-14

**Authors:** Sewunet Admasu Belachew, Lisa Hall, Linda A. Selvey

**Affiliations:** 1 School of Public Health, The University of Queensland, 288 Herston Rd, Herston, QLD 4006 Australia; 2 School of Pharmacy, Faculty of Medicine and Health Sciences, University of Gondar, Gondar, Ethiopia

**Keywords:** Antibiotics dispensing, Community pharmacy, Non-prescription, Over the counter, Sub-Saharan Africa

## Abstract

**Background:**

The development of antimicrobial resistance, which is partially attributable to the overuse and/or misuse of antibiotics in health care, is one of the greatest global public health challenges. In Sub-Saharan African (SSA) countries, non-prescribed dispensing of antibiotics in community drug retail outlets (CDROs) has been flagged as one of the contributing factors for the widespread misuse of antibiotics in the community.

**Objective:**

The current review aimed to estimate the proportion of non-prescription antibiotics requests or consultations that resulted in provision of antibiotics without a valid prescription among CDROs in SSA region, and describe the type of antibiotics dispensed.

**Methods:**

A literature search was conducted using PubMed, CINAHL, Scopus and Google Scholar. We also searched reference lists of relevant articles. Random effect model meta-analysis was employed to determine the pooled proportion of over the counter sale of antibiotics. Subgroup and meta-regression was undertaken to explore the potential cause of heterogeneity in effect size across studies.

**Results:**

Of 671 total citations retrieved, 23 met the inclusion criteria (seven cross-sectional questionnaire-based surveys and 16 cross-sectional client-based studies). The overall pooled proportion of non-prescription antibiotics requests or consultations that resulted in supply of antibiotics without prescription was 69% (95% CI 58–80). Upper respiratory tract infections and/or acute diarrhoea were the most frequently presented case scenarios, and amoxicillin and co-trimoxazole were the most frequently dispensed antibiotics to treat those symptoms.

**Conclusions:**

Non-prescribed dispensing of antibiotics was found to be a common practice among CDROs in several SSA countries. Ease of access to and overuse of antibiotics can potentially accelerate the emergence of resistance to antibiotics available in the region. Our review highlights the need for a stringent enforcement of existing policies and/or enacting new regulatory frameworks that would regulate antibiotic supply, and training and educational support for pharmacy personnel (e.g. pharmacists, pharmacy assistants) regarding judicious use of antibiotics and the importance of antimicrobial stewardship.

## Introduction

The development of antimicrobial agents signifies one of the most significant attainments of modern medicine in the past century [1]. The total global antibiotics consumption increased by 65% (21.1 to 34.8 billion daily defined doses) in just 15 years (2000–2015), also projected to be much higher in 2030 [2]. The increase was primarily driven by increased consumption in low and middle-income countries (LMICs) [2, 3]. With growing economies and better access to pharmaceuticals, the rate of antibiotics consumption in many LMICs has now become comparable to or is surpassing those of high income countries [2, 4]. The rise of antibiotic consumption in Sub-Saharan African (SSA) countries is not different from other LMICs, and it may be worse because the region has one of the highest infectious disease burdens worldwide [5]. This high consumption of antibiotics, with or without prescription, has become a growing public health concern as it has been strongly implicated in the development of antimicrobial resistance (AMR) [6, 7].

While the causes of the development of AMR are complex and multifaceted, overuse and/or misuse of antibiotics in health care are among the main contributory factors [8]. It is estimated that by 2050, nearly 10 million deaths worldwide will be attributed to antimicrobial resistant infections and would cost the world up to USD 100 trillion if action is not taken to counter this crisis [9]. The greatest impact of AMR on the population health and health care cost would be in developing countries [10], particularly in SSA nations [11, 12]. This can be attributed to the relatively poor healthcare systems coupled with weak economies [4]. The full extent of AMR in LMICs countries is difficult to quantify based on the available studies, as this may be an underestimation due to lack of effective surveillance and reporting.

About 80% of antibiotics consumed worldwide are used in outside of the hospital settings [13]. It is estimated that over 50% of antibiotics worldwide are purchased privately from pharmacies or in the informal sector from street vendors often without prescriptions [14]. Misuse/overuse of antibiotics in several LMICs is facilitated by over the counter (OTC) supply of antibiotics on patient demand and/or suggestion by dispensers, where identifying individuals who really need antibiotics is a challenge [4, 15]. This unregulated non-prescription access of antibiotics has been reported as a major contributor to the emergence of AMR [16, 17]. Despite a legal framework in many countries in the developing world that prohibits dispensing antibiotics without a valid medical prescription, the OTC sales of antibiotics is frequent [16], and community drug retail outlets(CDROs) are the primary source of non-prescribed antibiotics [18, 19]. CDROs is a collective term that includes community pharmacies, drug stores or shops, rural drug vendors, and accredited drug dispensing outlets (ADDOs) available in the SSA region. Studies reported a range of antibiotics being dispensed without prescription in CDROs, but the predominant class of antibiotics dispensed differs from place to place [20, 21]. Identifying the frequently dispensed antibiotics in a certain region could enable targeted actions. Most of the CDROs in SSA simply provide antibiotics whenever demanded with very little history taking and counselling [22].

Given the above, an emphasis on antimicrobial stewardship efforts in CDROs is important. To date, two reviews [23, 24] have been published that explored the non-prescribed sale of antimicrobial agents globally, and in developing countries. However, findings from the above review may not be generalisable or informative to SSA countries. One review included only three studies from SSA countries, and both reviews were limited to only simulated client/case scenario based studies and articles published up to 2017. Hence, there is a need for an updated review and analysis of current evidence in SSA countries. Through this review we hope to generate strong and informative evidence about the problem in the region, which in turn, will enable planning of further investigation and inform efforts to curb AMR. Therefore, the aims of this systematic review and meta-analysis are to: summarise existing research and generate a strong combined evidence about the magnitude of non-prescription dispensing of antibiotic agents in CDROs of SSA countries and describe the type of antibiotics dispensed and case scenarios for which antibiotics were sought.

## Methods

The review was conducted in accordance with the Preferred Reporting Items for Systematic Reviews and Meta-Analyses (PRISMA) guideline [25], and the study protocol was registered on PROSPERO (CRD42020173317).

### Data sources and search strategy

We searched PubMed, CINAHL, Scopus and Google Scholar databases for studies that determined the extent of dispensing antibiotics without a valid prescription among CDROs in SSA countries. The keywords used in the search strategy were: (“Antibiotic” OR “anti-bacteria” OR “anti-microbe”) AND (“Dispense” OR sale* OR practice OR over the counter OR non-prescription OR “prescription” OR “without prescription” OR “Self-prescribe” OR “self-treatment” OR “self-medication”) AND (“Community Pharmacy” OR “Drug store/shop” OR “private pharmacy” OR “Community Pharmacy professionals” OR “Druggist/Pharmacy technicians” OR “drug/medicine vendor/personnel”). These were tailored to each database. Searches were restricted to studies undertaken in SSA countries. The search included articles published in English from the inception of each databases until the second week of March 2020.

Complementary searches (including forward and backward citation searches of included articles) were conducted to further locate eligible articles that were not identified in the databases search. Details on search terms and the number of records identified are provided in Additional file 1.

### Eligibility screening

The articles identified were exported from Endnote X9 to a screening tool called COVIDENCE to identify articles to be included. All titles, abstracts and full texts were independently screened by two reviewers to identify those that met the inclusion criteria, and differences were resolved through discussion between the two reviewers.

**Table:** 

Inclusion criteria	Exclusion criteria
Simulated client studies, onsite observation surveys or mixed methods studies, conducted in a CDRO setting, reporting the proportion of dispensing of antibiotics without a prescription Studies conducted in a CDRO setting involving interviewing pharmacy staffs and generating a report about a recent transaction made Studies involving exit interviews conducted with clients as they left retail outlets and reporting data on the OTC sale of antibiotics	Articles conducted in hospital or veterinary pharmacy settings Studies that were published in languages other than English Studies that explored knowledge and attitudes of pharmacists (or the public) about antibiotics but lacked data on the magnitude of non-prescribed sale of antibiotics Abstracts without the full text available for retrieval, and reviews, conference proceedings, letter to editor and meeting notes Pharmacy staffs (or public) opinion surveys on the supply and/or sources of non-prescribed antibiotics Studies not undertaken in SSA

### Quality assessment of the included studies

We employed the Joanna Briggs Institute’s critical appraisal checklist for prevalence studies to assess the quality of included studies [26]. Two independent reviewers performed the quality assessment of the studies.

### Data extraction

For all included full text articles, using a customised spreadsheet, one reviewer (SAB) extracted detailed information about the study characteristics and key findings (including author name, publication year, study country, study design, setting (e.g. pharmacy or drug store/shop or drug vendor), type of dispensers and key findings (number of pharmacy encounters, number of visits resulted in dispensing of antibiotics without valid prescription, types of antibiotics dispensed, types of disease/symptom presented and for which antibiotics were sought). A second reviewer (LH) checked the extracted data and no discrepancies were found. Definition of terms in the review are included in Additional file 2.

### Statistical analysis

Meta-analyses of the proportions of non-prescription dispensing of antibiotics were conducted using STATA version 16.0 (StataCorp. 2019.STATA version 16.0 Stata Statistical Software: Release 16. College Station, TX: StataCorp LLC). The proportion of non-prescription antibiotic requests, visits or transactions that resulted in provision of antibiotics in the absence of a valid prescription was used as an estimate of the level of non-prescription dispensing of antibiotics at CDROs. To account for heterogeneity of proportions across studies, a random effects model based on the DerSimonian and Laird approach was employed to determine the pooled estimate (with 95% confidence interval). The degree of heterogeneity across studies was estimated using I-squared (I^2^) statistics. Sub-group analysis was determined by study year, region, and number of CDROs, number of visits, and number of cases scenarios utilised. Meta-regression was used to identify and explain the potential causes of the observed heterogeneity using the following moderators: study approach, techniques used to sample CDROs (convenient, census, random), number of visits, and number of CDROs, region, and number of case scenarios used in each investigation. Funnel plot (and Egger’s test) were used to test for publication bias. We did sensitivity analysis to investigate the influence of each individual study on the overall meta-analysis summary estimate.

## Results

### Characteristics of the included studies

**Fig. 1:**
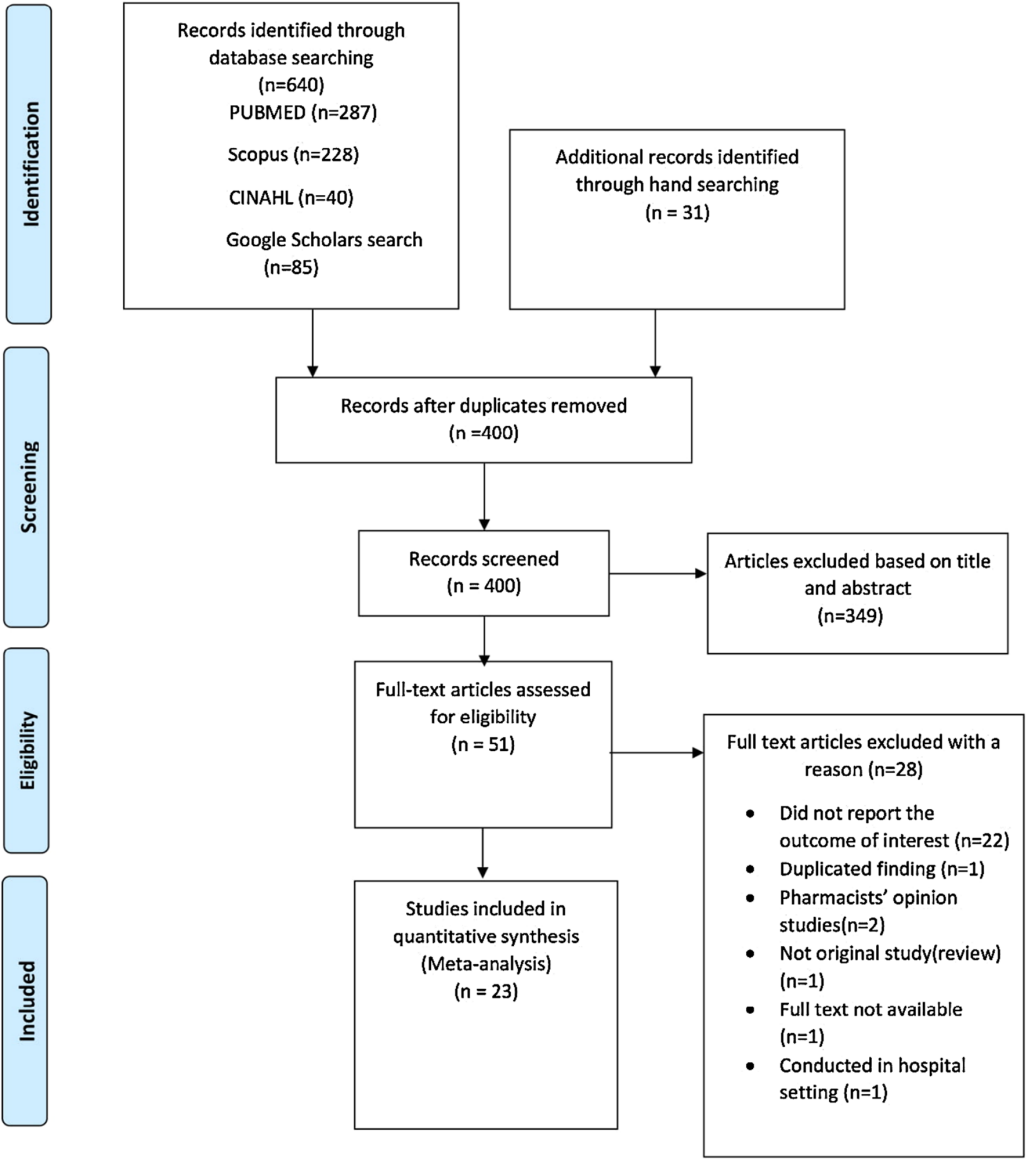
Flow diagram showing selection of eligible studies for inclusion in systematic review and meta-analysis

A search of the four databases, and reference lists or citations check yielded 671 search results. After eliminating the duplicates and excluding studies based on their abstracts or through examining their full text, 23 were identified as eligible for inclusion (Fig. 1). All the studies were cross-sectional and conducted in more than one CDROs (ranging from 6 to 306 CDROs). Sixteen out of 23 articles employed a simulated client [20, 27–41]. All included studies were conducted in SSA and most were from Ethiopia (n = 9) [28, 29, 32–34, 36, 37, 42, 43] and Tanzania (n = 5) [30, 35, 38, 40, 44]. Seventeen out of the 23 articles specifically described the type of CDROs investigated. These included, 298 pharmacies, 627 drug stores/shops, 14 rural drug outlets, and 398 ADDOs across seventeen localities in nine countries. Only four studies recorded who the dispenser on duty was. The total client encounters or visits across 23 articles was 4195. The number of visits/transactions in a single study ranged from 58 to 780 [43, 44].

Different studies employed a variety of approaches to assess the practice of antibiotics dispensing from CDROs. These included, symptom-based approaches in which clients request treatment or CDROs consultations by presenting clinical case scenarios or disease symptoms, and a direct product request for a specific antibiotic. Eighteen studies reported respiratory tract infection and/or acute childhood diarrhoea as the primary case scenarios for consultation and/or direct antibiotic requests [20, 27–39, 41, 44–46]; of which, ten studies only employed a symptom-based approach [20, 27–29, 33, 35, 37, 39, 40, 45]. Other case scenarios with symptoms of urethral discharge, vaginal discharge, injury/wound, and urinary tract infection were also used, but less commonly. Seven studies utilised both a symptom-based approach and direct product request [30–32, 34, 36, 38, 41]. Various classes of antibiotics including Penicillins, Sulphonamides, Nitro-imidazoles, Fluoroquinolones, and Macrolides were reported to be dispensed across studies (Table 1).

**Table 1:** Characteristics of the included studies in this systematic review and meta-analysis

Articles	Study locations	Data collection method	Case scenarios that resulted in antibiotics supply	Number of drug retail outlets visited	Number of visits/encounters/transactions	Encounters in which antibiotics were sold without prescriptions (%)	Main classes of antibiotics dispensed
Simulated client, acute childhood diarrhoea							
Abegaz et al. [28]	Ethiopia	Simulated client	Acute childhood diarrhoea	113	113	51	Sulphonamide, Nitro-imidazole
Berih et al. [27]	Sudan	Simulated client	Acute childhood diarrhoea	63	63	67	Sulphonamide, Nitro-imidazole
Mengistu et al. [37]	Ethiopia	Simulated client	Acute childhood diarrhoea	105	105	87	Sulphonamide
Simulated client, acute childhood diarrhoea plus adult upper respiratory infections (URTIs)							
Ayele et al. [29]	Ethiopia	Simulated client	Acute childhood diarrhoea and URTIs in adult	22	44	64	Sulphonamide, Nitro-imidazole, Penicillin, Macrolide Fluoroquinolone,
Erku DA et al. [33]	Ethiopia	Simulated client	Acute childhood diarrhoea and URTIs in adult	50	100	86	Sulphonamide, Nitro-imidazole, Penicillin, Macrolide, Fluoroquinolone Cephalosporin
Simulated client, acute childhood diarrhoea plus other conditions in all ages							
Nyazema et al. [39]	Zimbabwe	Simulated client	Acute childhood diarrhoea, vaginal discharge and itching, urethral discharge	87	184	8	Penicillin, Sulphonamide, Tetracycline, Fluoroquinolone
Koji et al. [36]	Ethiopia	Simulated client	Common cold, acute childhood diarrhoea, childhood pneumonia, meningitis, critically sick, then each case was followed by different antibiotics request	262	262	63	Penicillin, Macrolide, Sulphonamide, Nitro-imidazole, Cephalosporin, Aminoglycoside,
Minzi et al. [38]	Tanzania	Simulated client	Cough, headache and childhood diarrhoea, left hip injury, fever and childhood diarrhoea, vomiting and diarrhoea, persistent cough, yellowish urethral discharge with a bad smell, then each case was followed by different antibiotics request	145	145	69	Penicillin, Fluoroquinolone,
Simulated client, adult acute diarrhoea plus other conditions							
Bahta et al. [20]	Eritrea	Simulated Client	Acute watery diarrhoea and urinary tract infection(UTI)	84	153	88	Fluoroquinolone Sulphonamide, Penicillin, Tetracycline, Nitro-imidazole
Horumpende et al. [35]	Tanzania	Simulated client	Unspecified fever, cough, acute diarrhoea, pain during urination	39	39	92	Penicillin, Macrolide, Cephalosporin, Sulphonamide, Nitro-imidazole,, Fluoroquinolone
Damisie et al. [32]	Ethiopia	Simulated client	Acute diarrhoea, sore throat, UTI	18	54	87	Fluoroquinolone Sulphonamide, Penicillins Nitro-imidazole,, Macrolide
Simulated client, URTIs plus antibiotics product request in all ages							
Erku et al. [34]	Ethiopia	Simulated client	URTIs and amox-clav request	58	116	82	Penicillin Macrolides, Cephalosporin, Fluoroquinolone
Wafula et al. [41]	Kenya	Simulated client	Acute respiratory infection followed by amoxicillin request	200	200	87	Penicillin
Chikowe et al. [31]	Malawi	Simulated client	Direct product request(amoxicillin) (if asked, clarified as flu or cold)	45	45	93	Penicillin
Chalker et al. [30]	Tanzania	Simulated client	Cough and breathing difficulty and fast breathing with harsh noise; cough and runny nose, cough and runny nose then “co-trimoxazole (septrin) request	306	306	58	Sulphonamide
Simulated client, adult sexually transmitted infections							
Viberg et al. [40]	Tanzania	Simulated client	Vaginal discharge and itching, urethral discharge	94	251	53	Tetracycline, Penicillin, Sulphonamide, Macrolides, Fluoroquinolone, Nitro-imidazole,
Questionnaire based surveys, observation while pharmacy staffs are dispensing							
Ahiabu et al. [47]	Ghana	Observation at the pharmacies	N/A	6	307	91	Penicillin, Nitro-imidazole, fluoroquinolone, Sulphonamide, Tetracycline, Cephalosporin, Macrolide
Abula [42]	Ethiopia	Observation at the pharmacies	N/A	12	58	31	Penicillin, Sulphonamide
Questionnaire based surveys, onsite customers interview about their antibiotic purchase							
Elong Ekambi et al. [48]	Cameron	Interviewing clients at Pharmacies	N/A	7	402	47	N/A
Gebrekirstos et al. [43]	Ethiopia	Interview of clients at the pharmacies	N/A	14	780	38	NA
Questionnaire based surveys, dispensers asked about their antibiotic transaction (supplied for URTIs plus other conditions)							
Mbonye et al. [46]	Uganda	Interviewing the pharmacy staff on duty about recent sale	Childhood illness (URTIs, diarrhoea)	170	170	94	Penicillin, Sulphonamide, tetracycline, aminoglycoside
Kalungia et al. [45]	Zambia	A structured Interviewer administered questionnaire using simulated case scenario	Childhood respiratory tract infection and UTI	73	146	71	Penicillins, Sulphonamide, Nitro-imidazole
Questionnaire based surveys, customers’ requested about their antibiotic purchase when they left the CDROs							
Mboya et al. [44]	Tanzania	Interviewing clients at exit of the pharmacies	URTIs, UTI, diarrhoea/diarrhoea and vomiting, wound/abscess, urinary tract infection, abdominal pain, tonsillitis, asthma, fever, gastric ulcer,post-surgery, allergy, pneumonia, weeping eye, typhoid	12	152	76	Penicillin, Nitro-imidazole, Fluoroquinolone, Macrolide, Cephalosporin, Sulphonamide

NA, not available

### Quality assessment of the included studies

All the included studies were assessed for quality and risk of bias on nine criteria of Joanna Briggs Institute Critical Appraisal Checklist for Prevalence Studies. These criteria were: description of study settings and subjects, sample frame and sampling technique appropriateness, sample size adequacy, statistical analysis, method validity, data analysis coverage of all identified sample and adequacy of response rate, and whether the outcome variables were measured in reliable way. The majority of the studies (n = 18) met seven or more of the nine Joanna Briggs Institute appraisal checklist criteria for prevalence studies. Nearly half of the included studies did not describe the study subjects and the settings in detail. While all studies clearly outlined their research questions, eight studies did not justify the sample size used in their studies, and ten articles used convenience sampling technique to recruit the CDROs, which may affect generalisability. Details of the quality assessment for the included studies provided as an Additional file 3.

### Meta-analysis of the dispensing of antibiotics without a prescription

**Fig. 2:**
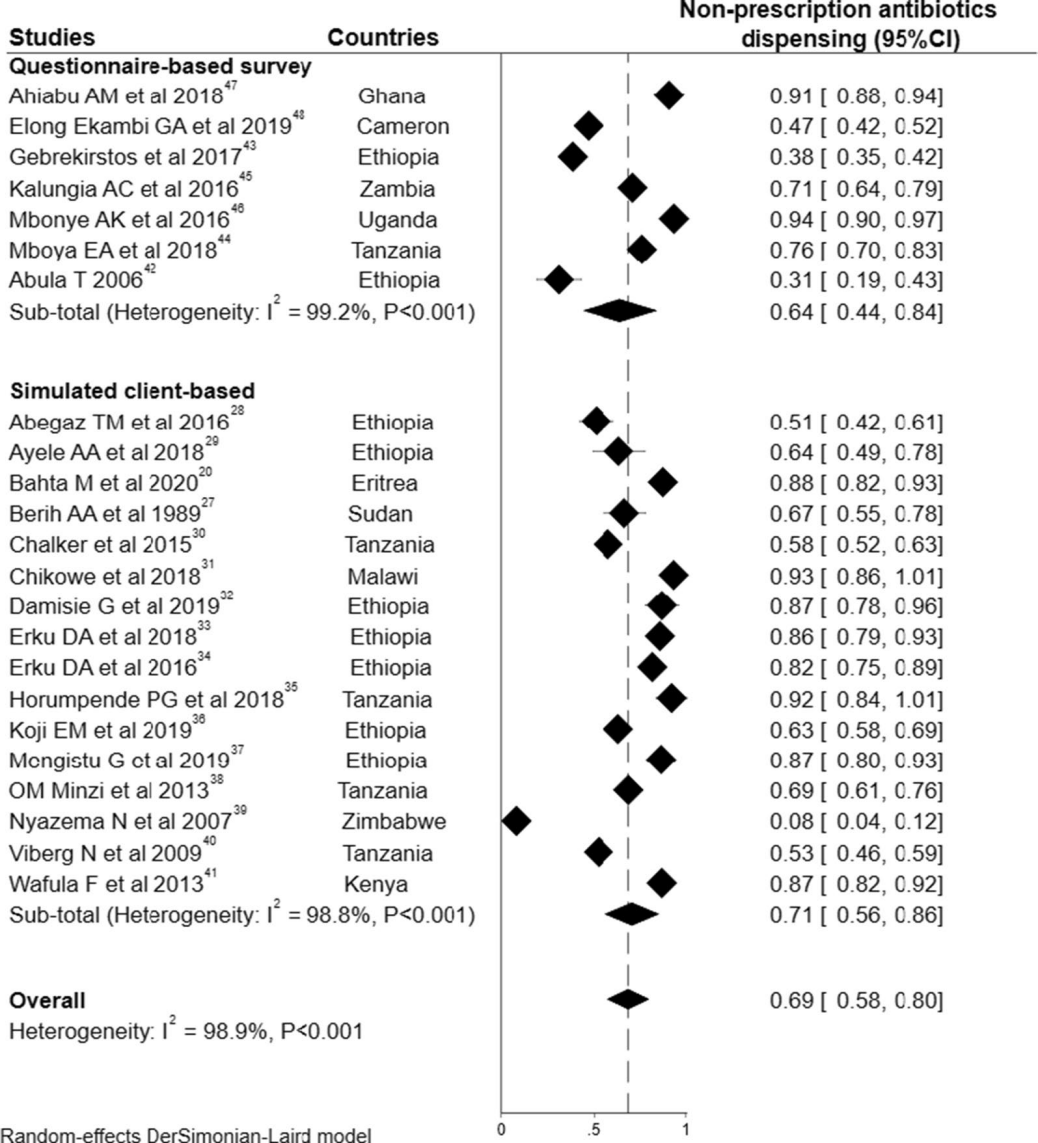
Meta-analysis for the overall pooled proportion of non-prescription antibiotics dispensing, n = 23 studies

The overall pooled estimate of non-prescription dispensing of antibiotics was 69% (95% CI 58–80) of antibiotic requests. The pooled estimate for simulated client-based studies was slightly higher 71% (95% CI 56–86) than the pooled estimate for cross-sectional questionnaire-based surveys 64% (95% CI 44–84). Considerable heterogeneity was noted on the pooled estimate of OTC sale of antibiotics (I^2^ = 98.9%, *P* < 0.001). The proportion of requests where non-prescribed antibiotics were sold ranged from 8% in Zimbabwe [39] to 94% in Uganda [46] (Fig. 2).

Sensitivity analysis was conducted by omitting each study and the exclusion of no single study significantly altered the combined estimate. In addition, there was no significant difference in the overall proportion of non-prescription antibiotics dispensing (70% (95% CI 62–78) when removing outlier studies (Additional file 4). Publication bias was assessed using a funnel plot (with Egger’s test), and statistical tests failed to show evidence of publication bias for included studies (Egger’s test: *P* = 0.667) (Additional file 5).

### Pooled estimates of non-prescribed sale of antibiotics by country

Several studies were undertaken only in either Ethiopia (n = 9)[28, 29, 32–34, 36, 37, 42, 43] or Tanzania (n = 5)[30, 35, 38, 40, 44], and the overall pooled proportion of antibiotic requests or consultations resulted in the supply of antibiotics without prescription for those countries were 66% ((95% CI 50–81), I^2^ = 97.9%, *P* < 0.001) and 69% (( 95% CI, 56–82), I^2^ = 94.6%*, P* < 0.001), respectively (Fig. 2).

### Subgroup meta-analysis of the proportion of non-prescription dispensing of antibiotics

A marked variation in pooled proportion of consultations where antibiotics were prescribed was noted across studies published from years 2000–2015 compared to 2016–2020, with estimate of 51% (95% CI 23–79) and 76% (95% CI 65–87), respectively. However, the difference in the pooled estimates between studies with different years of publication was not statistically significant (*P* = 0.066). There was also no difference in pooled estimates between studies using either single or multiple case scenarios (*P* = 0.235) (Table 2). Due to the high heterogeneity, we looked at the potential sources of heterogeneity through meta-regression. Of the potential sources of heterogeneity investigated, the variation in the pooled estimates, between studies with different years of publication and between studies with different numbers of patient visits, had *P* value less than 0.1 to explain the observed heterogeneity in effect size. These two covariates were taken to the multivariate meta-regression and yielded a significant multivariate model (*P* = 0.02) that explained 26.6% of between study variation.

**Table 2:** Subgroup meta-analysis of the proportion of non-prescription dispensing of antibiotics

Subgroups	Non-prescription dispensing of antibiotics(95% CI)	Difference in estimate between categories (*P* value)	Number of studies	Heterogeneity
Study publication year ^a^				
2016–2020	0.76 (0.65–0.87)	*P* = 0.066	16	I^2^ = 98.3%, *P* < 0.001
2000–2015	0.51 (0.23–0.79)		6	I^2^ = 99.3%, *P* < 0.001
< 2000	0.67 (0.54–0.77)		1	NA
Region				
East Africa	0.69 (0.57–0.81)	*P* = 0.66	21	I^2^ = 98.8%, *P* < 0.001
Central Africa	0.47 (0.42–0.52)		1	NA
West Africa	0.91 (0.87–0.94)		1	NA
Case scenarios				
Multiple**	0.71 (0.53–0.88)	*P* = 0.235	13	I^2^ = 99.0%, *P* < 0.001
Single*	0.76 (0.64–0.89)		6	I^2^ = 95.9%, *P* < 0.001
Not specified	0.52 (0.22–0.82)		4	I^2^ = 99.5%, *P* < 0.001
Number of visits***				
≤ 146	0.74 (0.65–0.83)	*P* = 0.320	12	I^2^ = 92.9%, *P* < 0.001
> 146	0.64 (0.46–0.82)		11	I^2^ = 99.4%, *P* < 0.001
Number of outlets***				
≤ 63	0.71 (0.57–0.86)	*P* = 0.603	12	I^2^ = 98.5%, *P* < 0.001
> 63	0.66 (0.48–0.85)		11	I^2^ = 99.2%, *P* < 0.001

NA, not applicable

^a^The World Health Organisation issued the global strategy for containment of antimicrobial resistance (AMR) in 2001, and global action plan to control AMR in 2015, categorised studies based on year using this fact

^**^Studies used more than one case scenario (includes; diarrhoea, URTI, vaginal discharge, urethral discharge, UTI, wound/abscess, injury, typhoid, post-surgery etc.)

^*^Studies used either diarrhoea or respiratory tract infection case scenarios

^***^Median was taken to set cut-off point for categorisation

### Proportion of non-prescribed antibiotics dispensed by symptom group

The overall pooled proportion of non-prescription antibiotic consultations that resulted in supply of antibiotics without prescription among clients presenting with acute childhood or watery diarrhoea alone was 60% (95% CI 33–86) (Table 3). The proportion of non-prescription sale of antibiotics for clients with this symptom ranged from 9% (95% CI 4–18) in a study done in Zimbabwe [39] to 89% (95% CI 67–97) in an Ethiopian study [32]. Co-trimoxazole was the most frequently antibiotic suggested and provided for the management of acute diarrhoea. The pooled proportion of non-prescription supply of antibiotics for patients with symptoms of upper respiratory tract infections (URTIs) with or without a direct antibiotics request was 84% (95% CI 74–94). Amoxicillin was the most commonly requested and recommended antibiotics to manage the symptoms of URTIs.

**Table 3:** Pooled estimates of the proportion of antibiotics dispensed without prescription based on disease/symptom category

Types of symptom	Pooled estimate %(95% CI)	Heterogeneity	Number of studies
Acute diarrhoea	60 (33–86)	I^2^ = 98.0%, *P* < 0.001	7 [27–29, 32, 33, 37, 39]
Upper respiratory tract infections with or without a direct antibiotics request	84 (74–94)	I^2^ = 92.1%, *P* < 0.001	6 [29–31, 33, 34, 41]
Sexually transmitted infections (STIs)	25 (21–28)	I^2^ = 0.0%	2 [39, 40]
Acute diarrhoea and Urinary tract infection	88 (81–92)	NA	1 [20]
Urinary tract infection (UTI)	94 (74–99)	NA	1 [32]
Sore throat	78 (55–91)	NA	1 [32]
Case not reported or reported but the magnitude for each symptom not identified	67 (52–83)	I^2^ = 98.9%, *P* < 0.001	10 [35, 36, 38, 42–48]

NA, not applicable

### Antibiotics dispensed without prescription

**Fig. 3:**
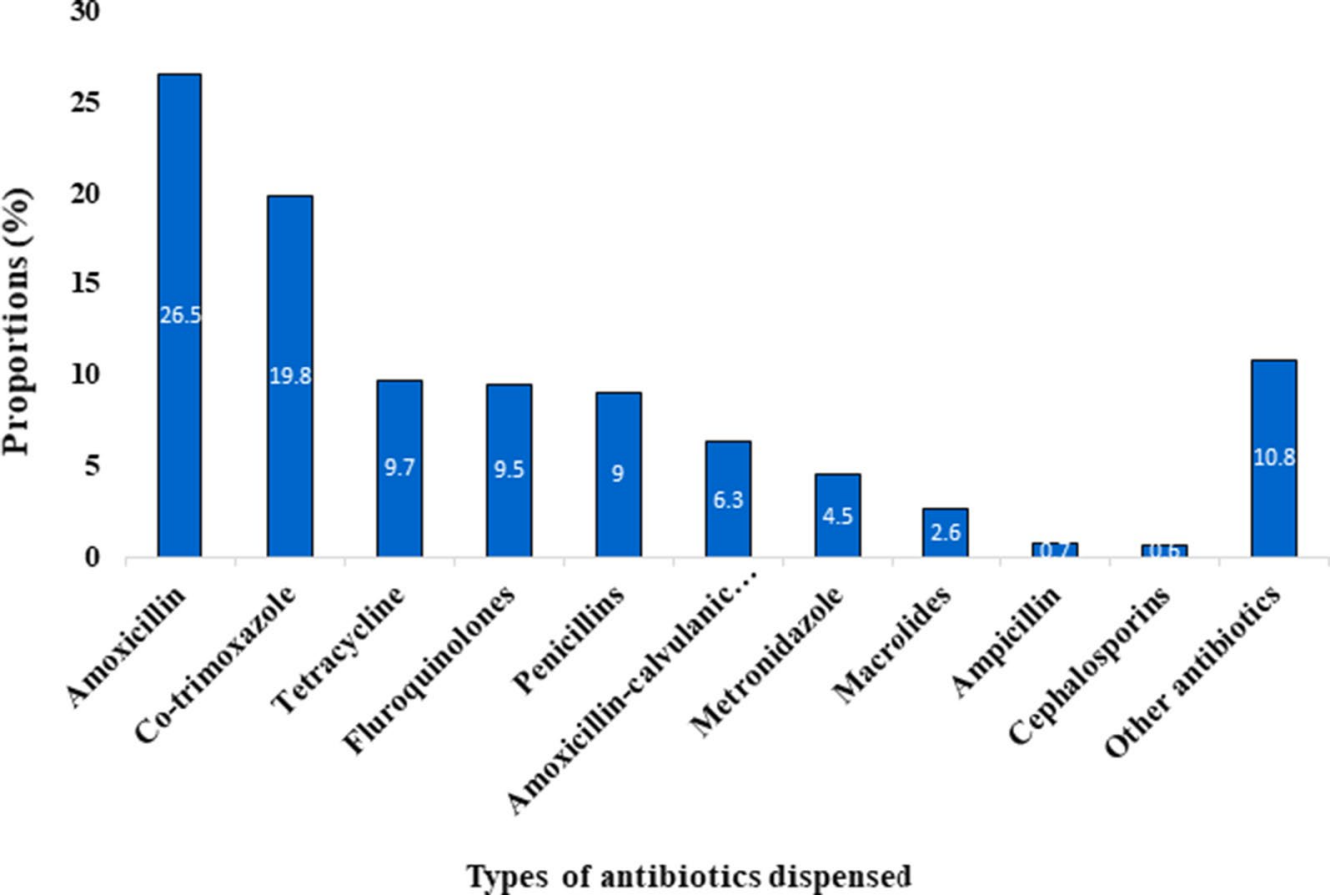
Proportion of antibiotics dispensed without prescription as reported in 13 studies

Thirteen studies reported the types of different antibiotics dispensed without prescription along with the respective frequencies [20, 28, 29, 31–34, 37, 40–42, 46, 47]. Overall, amoxicillin (26.5%) was the most commonly dispensed antibiotics followed by co-trimoxazole (19.8%%) and tetracycline (9.7%) (Fig. 3). While co-trimoxazole and amoxicillin-clavulanic acid were the most commonly supplied antibiotics in Ethiopia [26, 28, 29, 33–35, 38], doxycycline and ciprofloxacin were the primarily dispensed antibiotics in Tanzania [40].

## Discussion

We systematically collected, analysed, and synthesised findings of published researches on the supply of antibiotics without valid prescriptions. Our findings showed that non-prescribed dispensing of antibiotics among CDROs in SSA was very high, with more than two-thirds (69%) of the total antibiotics requests or consultations resulted in dispensing of antibiotics without a valid medical prescription. This review found that antibiotic agents can be purchased without prescription across a range of SSA countries. We noted a substantial heterogeneity in the proportion of non-prescription antibiotics supply between studies, ranging from 8% in Zimbabwe to 94% in Uganda. URTIs and acute diarrhoea were the most frequently presented case scenarios, and amoxicillin (26.5%) and co-trimoxazole (19.8%) were the most frequently dispensed antibiotics across the studies, respectively. Most of the antibiotics supplied for disease symptoms depicting acute URTIs or acute childhood diarrhoea were inappropriate since most of these clinical scenarios had cardinal symptoms suggestive of acute and simple viral causes.

Our findings showed the overall proportion of non-prescription sale of antibiotics was high and difference in prevalence of non-prescribed sale of antibiotics was noticed across countries. The high proportion of antibiotics dispensing in Uganda occurred even though this practice was illegal and the study authors speculated that the high rate of dispensing of non-prescribed antibiotics was due to weak enforcement of the regulations. This finding warrants investigation on where and how the system fails to enforce the law properly [46]. The lowest prevalence of non-prescription antibiotic sale was in Zimbabwe. This may be due to efforts commenced in 2000 to regulate for profit healthcare providers in the country. Hence, the country undertook strict enforcement of laws prohibiting the supply of antibiotics without prescription through establishing collaboration between the central and local regulatory authorities. The punishment for contravening this law includes revoking the professionals’ license [49, 50]. In 2015, the World Health Organization (WHO) adopted national action plans (NAPS) to combat antimicrobial resistance in which responsible use of antibiotics have been a core component [51]. Following the WHO’s urging for countries to develop their own NAPS, countries in SSA such as Ethiopia, Kenya, Tanzania and Zimbabwe developed their plans [52–55]. However, our review found that the non-prescription sale of antibiotics increased after 2015, suggesting that the implementation of the plan is weak. For instance, Ethiopia adopted its own NAPs early on 2015, but the current evidence shows the non-prescription sale of antibiotics is high [52]. Studies in Ethiopia identified a combination of reasons for the non-prescription sale in addition to weak regulatory system, including business interests and customers’ pressure [33, 56]. In Tanzania, ADDOs were launched in 2003 to improve accessibility of medicines, usually run by trained supervisors unlike pharmacies which are supervised by registered pharmacists [35]. Although ADDOs’ service improved accessibility, the impact in terms of augmenting the non-prescription antibiotic access have been shown by the current review through the included studies that evaluated ADDOs’ dispensing practice [30, 38, 44].

Our finding noted ease of acquisition of non-prescribed antibiotics from CDROs for the treatment of URTIs and childhood diarrhoea. In SSA region, respiratory tract infection and diarrhoeal diseases are among the leading causes of mortality [7]. However, most of the URTIs (such as common cold or flu) and acute childhood diarrhoea (such as non-dysentery or watery diarrhoea) are caused by viruses, are usually self- limiting and require symptomatic and/or fluid balance management only. A review in 2019 indicated rotavirus was a major causative agent of diarrhoea in African region [57]. The findings of our review could be one source of evidence to potentially explain the WHO report on surveillance of antibiotic consumption which indicated that 75% of antibiotics consumed in the African region were oral and parenteral antibiotics that mainly included amoxicillin, amoxicillin-clavulanic acid, ciprofloxacin, penicillin, co-trimoxazole and metronidazole [7].

The majority of the non-prescribed antibiotics identified in our review have been classified as lifesaving antibiotics by WHO (e.g. amoxicillin, co-trimoxazole, fluoroquinolones, macrolides, cephalosporines, tetracyclines) [58]. Amoxicillin, cotrimoxazole and ciprofloxacin are availiable and are affordable for the wider community in the SSA region [7]. However, the range of antibiotics availiable for treament of some specific disease conditions is very limited in the region. For instance, azithromicn is one of the limited availaible yet life saving drug to treat *Campylobacter* infections [59], especially when no other options are availiable. As a result of a repeated misuse or overuse, the emergence of resistance to these antibiotics could have a significant negative consequence on public health as the incidence of *Campylobacter* infections is high and increasing [60]. In our review, many of the antibiotics dispensed as OTC drugs were broad spectrum antibiotics. This might be because pharmacy staff opt to recommend broad-spectrum antibiotics as they will allow a greater range of pathogens to be covered thereby enhancing the likelihood of therapeutic success. However, to limit the risk of development of AMR and other adverse impacts, experts recommend narrower spectrum antibiotics for confirmed specific conditions. For example, while prescribed nitrofurantoin or trimethoprim are available first line narrow spectrum antibiotics to treat confirmed uncomplicated UTIs, patients with UTI symptoms frequently receive either fluoroquinolones or co-trimoxazole [61, 62]. Potential consequences of these choices include a greater risk of adverse drug reactions and selecting resistant bacteria. Broad-spectrum antibiotics can seriously disturb the normal intestinal flora and facilitate bacterial overgrowth with emergence of resistant microorganisms [8, 14, 63–65].

Stringent enforcement of laws prohibiting the non-prescription sale of antibiotics has been found to work well in containing the non-prescribed antibiotics provision in Zimbabwe, Chile, Colombia, Brazil, Mexico and Korea [50, 66–68]. WHO also suggested and emphasised enforcing prescription-only policies to reduce unnecessary use of antibiotics [69]. Countries included in the review have regulations that prohibit the non-prescription sale of antibiotics, but the sale of antibiotics without a valid prescription is mounting. Hence, our review highlights the need for ensuring stringent enforcement of laws and adherence to regulations to avert the reckless behaviour in relation to antibiotics dispensing without prescription at CDROs [70, 71].

Availability and accessibility of primary healthcare facilities in the SSA region is limited, and CDROs are located within communities they serve as the first point of contact for the public. As they are the main source of antibiotics for the wider population in the region [18, 19], they could play a key role in protecting antibiotics and educating customers about rational antibiotic use. CDROs’ active involvement in delivering information or consultations for visiting clients about appropriate antibiotics use and furnishing them with up-to-date information would be one cost-effective strategy to contain antibiotic misuse or over use [70]. In this regard, capacity building training of pharmacy staff about rational antibiotic use or dispensing and importance of antibiotic stewardship could be important. For instance, in 2018, a review done in developing countries noted that appropriately trained pharmacists can be part of the solution to overcome the global challenge of AMR and emphasised that training can enhance the role of pharmacy professionals in antibiotic stewardship [72]. It would be also worthwhile holding regular population awareness raising campaign to reduce intense public antibiotic demand and promote rational community antibiotic use. For instance, evidence from Europe showed that public antibiotic use awareness campaigns yielded a reduction in public antibiotic use of 6.5– 28.3% [73]. A randomised and controlled study in India also strengthened a point that public education about medicine use resulted a substantial improvement in rational use of medicines [74].

Our review highlights several areas that merit further study. First, many of the included studies were conducted in administrative centres /major towns and only a few studies included in our review have reported data from rural towns where the regulatory bodies’ inspection is assumed to be relatively less stringent. Evidence in SSA suggests that characteristics and practices of drug shops vary between rural and urban locations [22]. In addition, many of the included studies were not representative as they involved small local areas or convenience sampling of CDROs. Thus, there is a need for future research that explores the extent and dispensing behaviour of pharmacy staff in CDROs using representative samples and including CDROs from rural district administrations. Our review also highlights the need for mixed methods studies to examine the knowledge, beliefs and practice of pharmacy staff in relation to antibiotic use, along with a qualitative investigation of the reason behind selling of antibiotics without valid prescription in CDROs. Such studies produce more complete evidence about the problem and the potential causes and enables stakeholders to plan targeted interventions. One of the cornerstones of the WHO Global Action plan is the Antibiotic Stewardship Program [51]. Community pharmacy staff are ideal antibiotic stewards to contain the threat of AMR given their location and easy accessibility, but their potential is largely untapped [75]. Such research may provide insights to inform antimicrobial stewardship programs in CDRO settings.

### Strength and limitations

To the best of our knowledge and search, this systematic review and meta-analysis is the first of its kind to analyse and summarise the current literature regarding the extent of dispensing of antibiotics without valid prescriptions in the SSA region where the infection burden is disproportionately high. However, our review is not without limitations. Although we have utilised comprehensive search strategies to include client-based studies as well as other relevant surveys, it is possible that we may have missed some studies that are not indexed in the included databases or published in English. Second, although the estimates used random effects meta-analysis to account for heterogeneity between studies, there were significant heterogeneity in effect size across the studies. Furthermore, a lack of data from many SSA countries was also a limitation of the review.

## Conclusion

This review and meta-analysis provide a regional estimate of non-prescription sale of antibiotics among CDROs in SSA countries. Non-prescribed dispensing of antibiotics was found to be a common practice among CDROs in several SSA countries. Amoxicillin and co-trimoxazole were identified as the leading antibiotics sold without prescription. Upper respiratory tract infections and/or acute diarrhoea were the top reasons for which antibiotics were supplied. Ease of access to and overuse can potentially accelerate the emergence of antibiotic resistance to few yet lifesaving antibiotics available in the region. Our review highlights the need for stringent enforcement of existing policies and/or enacting new regulatory frameworks that would regulate antibiotic supply, and continuous training and educational support for pharmacy personnel (e.g. pharmacists, pharmacy assistants) regarding judicious use of antibiotics and the importance of antimicrobial stewardship.

## Supplementary Information

**Additional file 1.** Search terms and strategy.

**Additional file 2.** Definition of terms.

**Additional file 3.** Quality assessment of the included studies.

**Additional file 4.** Sensitivity analysis.

**Additional file 5.** Funnel plot to assess publication bias.

## Data Availability

All data generated or analysed during this study are included in this published article and its supplementary information files.

## Abbreviations

ADDOs: Accredited drug dispensing outlets
AMR: Antimicrobial resistance
CI: Confidence interval
CDROs: Community drug retail outlets
LMICs: Low and middle income countries
NAPs: National action plans
OTC: Over the counter
PRISMA: Preferred Reporting Items for Systematic Reviews and Meta-Analyses
STATA: Stata Statistical Software
SSA: Sub-Saharan Africa
USD: United States Dollar
URTI: Upper respiratory tract infection
UTI: Urinary tract infection
WHO: World Health Organization
