# Supplementary material for: Non-prescription dispensing of antibiotic agents among community drug retail outlets in Sub-Saharan African countries: a systematic review and meta-analysis
Source: Antimicrob Resist Infect Control. 2021 Jan 14;10:13. doi: 10.1186/s13756-020-00880-w (PMC7807893; doi:10.1186/s13756-020-00880-w)
Supplement: Supplementary file 1 — Additional file 1. Search terms and strategy. [file 13756_2020_880_MOESM1_ESM.docx]

| **Supplementary Material**  **SI: Search terms and strategy**  **Title:** **Non-prescription dispensing of antibiotic agents among community drug retail outlets in Sub-Saharan African countries: a systematic review and meta-analysis**  Main concepts from the research question that we used to search:   \| ­­­­­­­­­­­­­­­­­­­­­­­­­­­­ Population or participants or condition of interest \| Interventions/exposures \| Comparisons or control groups \| Outcome of interest \| \| --- \| --- \| --- \| --- \| \| - Community Pharmacy Professionals ( Pharmacists, druggists/pharmacy technicians) or Community drug retail outlets (Pharmacies, drug stores, rural drug vendors) \| Non-prescription dispensing of antibiotics \| No comparator \| - Magnitude of antibiotics dispensed with and/or without prescription in community retail outlets in SSA countries. \|   **Search terms**    **PubMed (287)**  ((((((((((((((((("Anti-Bacterial Agents"[Mesh]) OR "Anti-Bacterial Agents" [Pharmacological Action])) OR "Anti-Infective Agents"[Mesh])) OR Antibiotic* [tiab]) OR antimicrob*[tiab])) OR anti-bacteria* [tiab]) OR anti-infective* [tiab]))) AND ((((((((((((((((drugs, over the counter[MeSH Terms]) OR over the counter [tiab]) OR misuse* [tiab] OR overuse * [tiab] OR dispens* [tiab]) OR sale* [tiab]) OR self-medication[MeSH Terms]) OR self-medication* [tiab]) OR sale[MeSH Terms]) OR self-prescrib* [tiab]) OR suppl* [tiab]) OR non-prescri* [tiab]) OR without-prescription* [tiab]) OR with-prescription* [tiab] OR prescription* [tiab] OR practice [tiab]) OR non-judicious [tiab] OR non-OTC drug* [tiab] OR purchase* [tiab] OR distribut* [tiab] OR management* [tiab]) OR self-treatment* [tiab]) OR provision* [tiab]))) AND ((((((((((((((community pharmacies[MeSH Terms]) OR community-pharmac* [tiab]) OR drug-store* [tiab]) OR drug-shop* [tiab]) OR retail-outlet* [tiab]) OR private-pharmac* [tiab]) OR pharmacist[MeSH Terms]) OR community-pharmacist* [tiab]) OR druggist* [tiab]) OR drug-personnel* [tiab]) OR pharmacy-assistant* [tiab]) OR pharmacy-technician* OR drug-vendor* [tiab]) OR medicine-vendor* [tiab]) OR community pharmacy service[MeSH Terms]))) AND (((("Africa South of the Sahara"[Mesh]) OR ("Central Africa" OR Cameroon OR "Central African Republic" OR Chad OR Congo OR "Democratic Republic of the Congo" OR "Equatorial Guinea" OR Gabon OR "Sao Tome and Principe" OR "Eastern Africa" OR Burundi OR Djibouti OR Eritrea OR Ethiopia OR Kenya OR Rwanda OR Somalia OR "South Sudan" OR Sudan OR Tanzania OR Uganda OR "Southern Africa" OR Angola OR Botswana OR Eswatini OR Lesotho OR Malawi OR Mozambique OR Namibia OR South Africa OR Zambia OR Zimbabwe OR Africa, Western OR Benin OR "Burkina Faso" OR "Cabo Verde" OR "Cote d'Ivoire" OR Gambia OR Ghana OR Guinea OR Guinea-Bissau OR Liberia OR Mali OR Mauritania OR Niger OR Nigeria OR Senegal OR "Sierra Leone" OR Togo))))))  **Scopus (228)**  ( TITLE-ABS-KEY ( "antibacterial agent*"  OR  antibiotic*  OR  "antiinfective agent*"  OR  bacteriocid*  OR  antimicrob*  OR  antibacteria* ) )  AND  ( ( TITLE-ABS-KEY ( "over the counter"  OR  dispens*  OR  misuse*  OR  overuse*  OR  sale*  OR  "self medication*"  OR  "self prescrib*"  OR  suppl*  OR  "non prescri*"  OR  "without prescription*"  OR  prescription*  OR  practice*  OR  management*  OR  "self treatment*"  OR  provi* )  OR  TITLE-ABS-KEY ( "with prescription*"  OR  ''non-judicious''  OR  "non-OTC drug*"  OR  purchase*  OR  distribut* ) ) )  AND  ( ( TITLE-ABS-KEY ( "community pharmac*"  OR  "drug store*"  OR  "drug shop*"  OR  "retail outlet*"  OR  "private pharmac*"  OR  pharmacist*  OR  "community pharmacist*"  OR  druggist*  OR  "drug personnel*"  OR  "pharmacy assistant*"  OR  "pharmacy technician*"  OR  "drug vendor*"  OR  "medicine vendor*" )  OR  TITLE-ABS-KEY ( "community pharmacy service*" ) ) )  AND  ( ( TITLE-ABS-KEY ( "central africa"  OR  cameroon  OR  "central african republic"  OR  chad  OR  congo  OR  "democratic republic congo"  OR  "equatorial guinea"  OR  gabon  OR  "sao tome and principe"  OR  "eastern Africa"  OR  burundi  OR  djibouti  OR  eritrea )  OR  TITLE-ABS-KEY ( ethiopia  OR  kenya  OR  rwanda  OR  somalia  OR  "south sudan"  OR  sudan  OR  tanzania  OR  uganda  OR  angola  OR  "southern africa"  OR  botswana  OR  eswatini  OR  lesotho  OR  malawi  OR  mozambique  OR  namibia  OR  "south africa"  OR  zambia )  OR  TITLE-ABS-KEY ( zimbabwe  OR  "western africa"  OR  benin  OR  "burkina faso"  OR  "cabo verde"  OR  "cote d'ivoire"  OR  gambia  OR  ghana  OR  guinea  OR  "guinea bissau"  OR  liberia  OR  mali  OR  mauritania  OR  niger  OR  nigeria  OR  senegal )  OR  TITLE-ABS-KEY ( "Sierra Leone"  OR  togo ) ) )  **CINAHL(40)**  ( "antibacterial agent*" OR antibiotic* OR "antiinfective agent*" OR bacteriocid* OR antimicrob* OR antibacteria* ) AND ( "over the counter" OR dispens* OR misuse* OR overuse* OR sale* OR "self-medication*" OR "self prescrib*" OR suppl* OR "non prescri*" OR "without prescription*" OR prescription* OR practice* OR management* OR "self treatment*" OR provi* OR "with prescription*" OR ''non-judicious'' OR "non-OTC drug*" OR purchase* OR distribut* ) AND ( "community pharmac*" OR "drug store*" OR "drug shop*" OR "retail outlet*" OR "private pharmac*" OR pharmacist* OR "community pharmacist*" OR druggist* OR "drug personnel*" OR "pharmacy assistant*" OR ''pharmacy technician*'' OR "drug vendor*" OR "medicine vendor*" OR "community pharmacy service*" ) AND ( "central africa" OR cameroon OR "central african republic" OR chad OR congo OR "democratic republic congo" OR "equatorial guinea" OR gabon OR "sao tome and principe" OR "eastern Africa" OR burundi OR djibouti OR eritrea OR ethiopia OR kenya OR rwanda OR somalia OR "south sudan" OR sudan OR tanzania OR uganda OR angola OR "southern africa" OR botswana OR eswatini OR lesotho OR malawi OR mozambique OR namibia OR "south africa" OR zambia OR zimbabwe OR "western africa" OR benin OR "burkina faso" OR "cabo verde" OR "cote d'ivoire" OR gambia OR ghana OR guinea OR "guinea bissau" OR liberia OR mali OR mauritania OR niger OR nigeria OR senegal OR "Sierra Leone" OR togo )    **Google scholar(85)**  Targeted search using key terms  **Hand searching(31)**  Backward and forward citations search |
| --- | --- | --- | --- | --- | --- | --- | --- | --- |
