# Supplementary material for: Non-prescription dispensing of antibiotic agents among community drug retail outlets in Sub-Saharan African countries: a systematic review and meta-analysis
Source: Antimicrob Resist Infect Control. 2021 Jan 14;10:13. doi: 10.1186/s13756-020-00880-w (PMC7807893; doi:10.1186/s13756-020-00880-w)
Supplement: Supplementary file 2 — Additional file 2. Definition of terms. [file 13756_2020_880_MOESM2_ESM.docx]

**Definitions of terms:**

**Penicillins:** includes antibiotics mentioned as penicillins, penicillin V, benzyl penicillin forte, benzyl phenoxy penicillin, amoxicillin and phenoxy methyl penicillin; **Fluoroquinolones:** includes fluoroquinolones, ciprofloxacin, norfloxacin, levofloxacin; **Macrolides** includes azithromycin, erythromycin; **Cephalosporin** includes: cephalexin, cefixime, **Tetracyclines:** includes tetracycline, doxycycline. **Other antibiotics** (**N.B**: the list of antibiotics enclosed in bracket is to indicate that the frequencies were reported as a group): Tinidazole, ampiclox, nitrofurantoin, chloramphenicol, Rifampicin, Gentamicin, [amoxicillin and azithromycin], [ciprofloxacin and tinidazole and metronidazole], [co-trimoxazole and metronidazole].

**Community drug retail outlets (CDROs):** This is a collective name to include drug stores/shops, pharmacies, rural drug vendors and accredited drug dispensing outlets (ADDOs).

**Simulated client studies:** This is a method in which an individual acts as a real patient and simulates a set of symptoms or problems to evaluate a practice or service.

**Questionnaire based surveys**: This is a method in which either the dispensers were asked about their recent non-prescription antibiotics transactions or the customers were asked about the antibiotics they purchased as they left the CDROs or overt onsite observation by third party while dispensing.
