# Supplementary material for: Non-prescription dispensing of antibiotic agents among community drug retail outlets in Sub-Saharan African countries: a systematic review and meta-analysis
Source: Antimicrob Resist Infect Control. 2021 Jan 14;10:13. doi: 10.1186/s13756-020-00880-w (PMC7807893; doi:10.1186/s13756-020-00880-w)
Supplement: Supplementary file 3 — Additional file 3. Quality assessment of the included studies. [file 13756_2020_880_MOESM3_ESM.docx]

**JBI Critical Appraisal Checklist for Studies Reporting Prevalence Data**

| **No** | **Criteria** | **S1** | **S2** | **S3** | **S4** | **S5** | **S6** | **S7** | **S8** | **S9** | **S10** | **S11** | **S12** | **S13** | **S14** | **S15** | **S16** | **S17** | **S18** | **S19** | **S20** | **S21** | **S22** | **S23** |
| --- | --- | --- | --- | --- | --- | --- | --- | --- | --- | --- | --- | --- | --- | --- | --- | --- | --- | --- | --- | --- | --- | --- | --- | --- |
| 1 | Was the sample frame appropriate to address the target population? | N | Y | Y | Y | Y | Y | Y | Y | Y | Y | Y | Y | Y | Y | Y | Y | Y | Y | Y | Y | Y | Y | Y |
| 2 | Were study participants sampled in an appropriate way? | Y | N | Y | N | N | Y | N | Y | N | N | Y | Y | Y | N | Y | Y | Y | Y | Y | N | Y | Y | Y |
| 3 | Was the sample size adequate? | Y | N | N | Y | N | Y | N | Y | Y | Y | Y | Y | Y | Y | Y | Y | Y | Y | Y | U | Y | Y | N |
| 4 | Were the study subjects and the setting described in detail? | Y | Y | Y | Y | N | N | N | N | Y | Y | Y | Y | Y | N | Y | Y | Y | Y | Y | N | N | N | N |
| 5 | Was the data analysis conducted with sufficient coverage of the identified sample? | Y | Y | Y | Y | Y | Y | Y | Y | Y | Y | Y | Y | Y | Y | Y | Y | Y | Y | Y | Y | Y | Y | Y |
| 6 | Were valid methods used for the identification of the condition? | Y | N | Y | Y | Y | Y | Y | Y | Y | Y | Y | N | Y | Y | Y | N | Y | Y | Y | Y | Y | Y | N |
| 7 | Was the condition measured in a standard, reliable way for all participants? | Y | N | Y | Y | Y | Y | Y | Y | Y | Y | Y | N | Y | Y | Y | N | U | Y | Y | Y | Y | Y | N |
| 8 | Was there appropriate statistical analysis? : Yes | Y | Y | Y | Y | Y | Y | Y | Y | Y | Y | Y | Y | Y | Y | Y | Y | Y | Y | Y | Y | Y | Y | Y |
| 9 | Was the response rate adequate, and if not, was the low response rate managed appropriately? | Y | Y | Y | Y | U | Y | Y | Y | Y | Y | Y | Y | Y | Y | Y | Y | Y | Y | N | Y | U | Y | Y |
|  | Total score (max 9) | 8 | 5 | 8 | 8 | 6 | 8 | 6 | 8 | 8 | 8 | 9 | 7 | 9 | 7 | 9 | 7 | 8 | 9 | 8 | 6 | 7 | 8 | 5 |

**N:** no **Y:** yes **NA:** Not applicable **U:** unclear

**Studies (S):**

**S 1**: Management of children’s acute diarrhoea by community pharmacies in five towns of Ethiopia: simulated client case study

**S 2:** Treatment practices of households and antibiotic dispensing in medicine outlets in developing countries: The case of Ghana

**S 3:** Management of minor ailments in a community pharmacy setting: Findings from simulated visits and qualitative study in Gondar town, Ethiopia

**S 4**: Dispensing of antibiotics without prescription and associated factors in drug retail outlets of Eritrea: A simulated client method

**S 5**: Pharmacy dispensing practices for Sudanese children with diarrhoea.

**S 6**: What roles do accredited drug dispensing outlets play in facilitating access to antimicrobials? Results of a multi-method analysis

**S 7.** Amoxicillin Quality and Selling practice in urban pharmacies and drug stores of Blantyre, Malawi

**S 8**. Over the Counter Sale of Antibiotics at Drug Stores Found in Mizan-Aman Town, Southwest Ethiopia: A Cross-Sectional Simulated Client Visit Study

**S 9**: Knowledge, practices and attitudes on antibiotics use in Cameroon: Self-medication and prescription survey among children, adolescents and adults in private pharmacies

**S 10**: Non-prescribed sale of antibiotics for acute childhood diarrhoea and upper respiratory tract infection in community pharmacies: a 2 phase mixed-methods study

**S 11**: Extent of dispensing prescription-only medications without a prescription in community drug retail outlets in Addis Ababa, Ethiopia: a simulated-patient study

**S 12**: Non-prescribed antimicrobial use and associated factors among customers in drug retail outlet in Central Zone of Tigray, northern Ethiopia: a cross-sectional study

**S 13**: Prescription and non-prescription antibiotic dispensing practices in part I and part II pharmacies in Moshi Municipality, Kilimanjaro Region in Tanzania: A simulated clients approach

**S 14**: Non-prescription sale and dispensing of antibiotics in community pharmacies in Zambia

**S 15**: Practice of over-the-counter dispensary of antibiotics for childhood illnesses in Addis Ababa, Ethiopia: a simulated patient encounter study

**S 16:** Prescription for antibiotics at drug shops and strategies to improve quality of care and patient safety: a cross-sectional survey in the private sector in Uganda

**S 17**: Irrational use of antibiotics in the Moshi Municipality Northern Tanzania: a cross sectional study

**S 18**: Self-Reported and Actual Involvement of Community Pharmacy Professionals in the Management of Childhood Diarrhoea: A Cross-Sectional and Simulated Patient Study at two Towns of Eastern Ethiopia.

**S 19**: Low sale of antibiotics without prescription: a cross-sectional study in Zimbabwean private pharmacies

**S 20**: Application of basic pharmacology and dispensing practice of antibiotics in accredited drug-dispensing outlets in Tanzania

**S 21**: STI management in Tanzanian private drugstores: practices and roles of drug sellers

**S 22**: Availability and Dispensing Practices for antimalarial and antimicrobials in Western Kenyan Pharmacies

**S 23**: Assessment of the dispensing practice of drug retail outlets in selected towns, North West Ethiopia
