# Supplementary material for: Non-prescription dispensing of antibiotic agents among community drug retail outlets in Sub-Saharan African countries: a systematic review and meta-analysis
Source: Antimicrob Resist Infect Control. 2021 Jan 14;10:13. doi: 10.1186/s13756-020-00880-w (PMC7807893; doi:10.1186/s13756-020-00880-w)
Supplement: Supplementary file 4 — Additional file 4. Sensitivity analysis. [file 13756_2020_880_MOESM4_ESM.docx]

**Supplementary file**

**S2: Assessment of the influence of studies on overall estimate**

**Supplementary Fig : Senisitivity analysis**

| **Number of outlier studies selected** | **Pooled estimate excluding the outliers** |
| --- | --- |
| 8 | 70% (95%CI 62-78), I-squared=95.5%, *P*≤ 0.001 |
