# Supplementary figures and images for: Non-prescription dispensing of antibiotic agents among community drug retail outlets in Sub-Saharan African countries: a systematic review and meta-analysis
Source: Antimicrob Resist Infect Control. 2021 Jan 14;10:13. doi: 10.1186/s13756-020-00880-w (PMC7807893; doi:10.1186/s13756-020-00880-w)

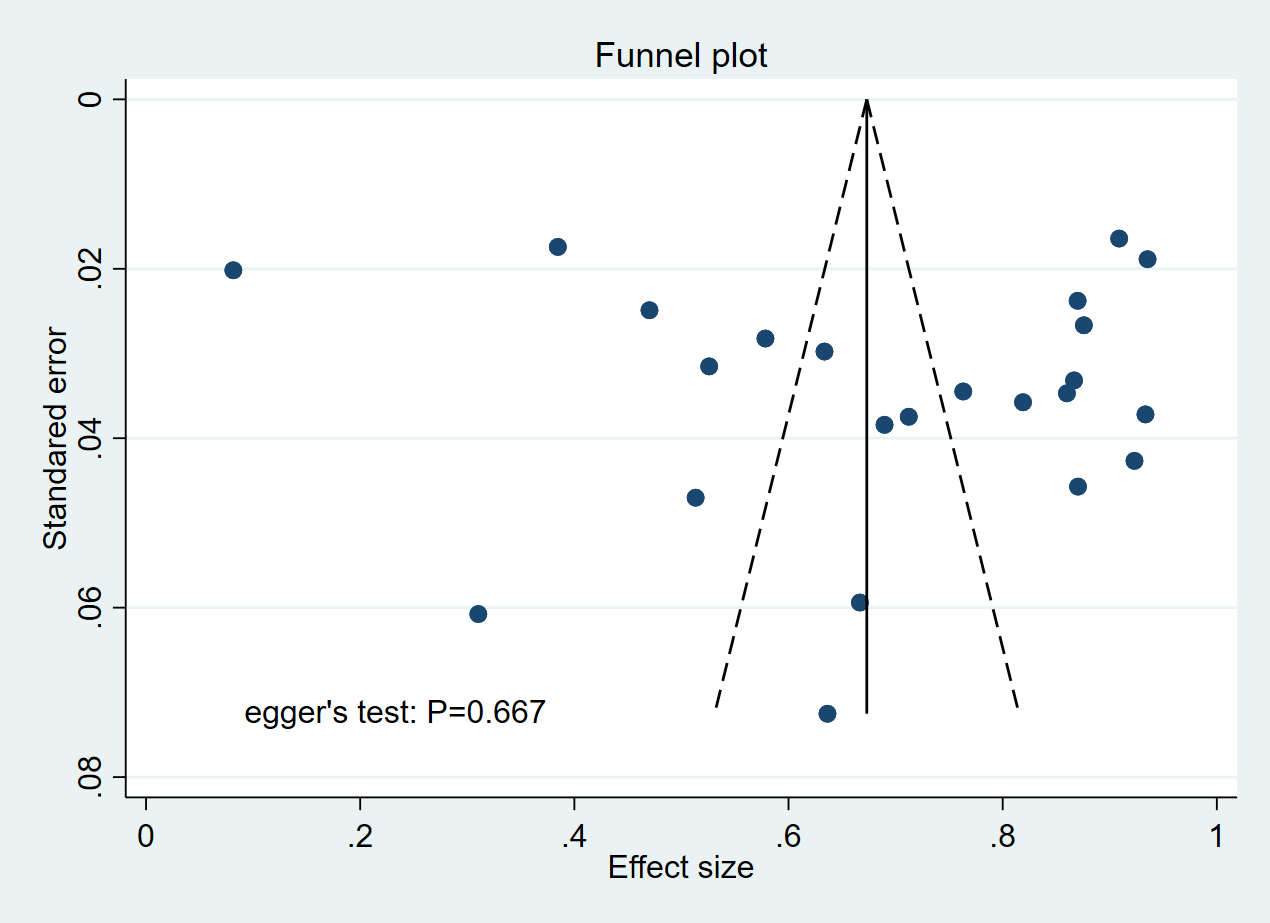

Supplement: Supplementary file 5 — Additional file 5. Funnel plot to assess publication bias. [file 13756_2020_880_MOESM5_ESM.tif]
